# Quantity Discrimination in Wolves (*Canis lupus*)

**DOI:** 10.3389/fpsyg.2012.00505

**Published:** 2012-11-16

**Authors:** Ewelina Utrata, Zsófia Virányi, Friederike Range

**Affiliations:** ^1^Department of Biology, University of HamburgHamburg, Germany; ^2^Messerli Research Institute, University of Veterinary Medicine ViennaVienna, Austria; ^3^Department of Cognitive Biology, University of ViennaVienna, Austria; ^4^Wolf Science CenterErnstbrunn, Austria

**Keywords:** numerical competence, Weber’s law, domestication, wolf

## Abstract

Quantity discrimination has been studied extensively in different non-human animal species. In the current study, we tested 11 hand-raised wolves (*Canis lupus*) in a two-way choice task. We placed a number of food items (one to four) sequentially into two opaque cans and asked the wolves to choose the larger amount. Moreover, we conducted two additional control conditions to rule out non-numerical properties of the presentation that the animals might have used to make the correct choice. Our results showed that wolves are able to make quantitative judgments at the group, but also at the individual level even when alternative strategies such as paying attention to the surface area or time and total amount are ruled out. In contrast to previous canine studies on dogs (*Canis familiaris*) and coyotes (*Canis latrans*), our wolves’ performance did not improve with decreasing ratio, referred to as Weber’s law. However, further studies using larger quantities than we used in the current set-up are still needed to determine whether and when wolves’ quantity discrimination conforms to Weber’s law.

## Introduction

Being able to discriminate between different quantities yields advantages for animals’ fitness and survival (Gallistel, 1990). For example, according to the optimal foraging theory, animals optimize their energy intake (Stephens and Krebs, 1986), and studies on different great apes (*Pan paniscus*, *Pan troglodytes*, *Gorilla gorilla*, *Pongo pygmaeus*, e.g., Hanus and Call, 2007) have shown that animals prefer the larger quantity of food if they are presented with a choice between two available food amounts. Another context where some rudimentary numerical competence provides an advantage is participation in intergroup conflicts over food, mates, and territory (Parker, 1974). Playback experiments in lions (*Panthera leo*; McComb et al., 1994) and chimpanzees (Wilson et al., 2001) have shown that animals adjust their cooperative behavior to a quantitative estimation of the opponent’s strength in comparison to that of their own group (Maynard Smith and Price, 1973; Maynard Smith and Parker, 1976).

In theory, animals can base their decisions in such contexts on the number of items, a quantity assessment, or some correlated perceptual features. While numbers are regarded as the product of counting (one-by-one), continuous (uncountable) quantities are the product of measurements, making the former accurate, and the latter approximate (Gallistel and Gelman, 2000). Moreover, up to a number of four items, the discrimination is also possible without actual counting but instead by relying on pattern recognition (also referred to as subitizing; Gallistel and Gelman, 2000). Finally, independent of number or quantity, the choice for one set of items over another one can be affected by perceptual features such as the size of a food pile or the surface area.

Researchers have used various methods to disentangle whether animals rely on numerical or quantitative information or instead on perceptual features to make their decisions. In particular there are two paradigms which have been used extensively: the two-box spontaneous choice and the violation of expectation paradigm. In the two-box spontaneous choice paradigm, animals are encouraged to choose between two quantities of either simultaneously or sequentially presented food items that can be visible or invisible at the time of choice. If the two sets of items are presented simultaneously, non-human animals may choose based on surface area or other correlated perceptual features rather than numerical properties (but see Brannon and Terrace, 2000 for possible controls). Instead, if the items of each set are consecutively presented and invisible during the choice, the subject never sees the entire content of either set, but instead must modify its representation of each set’s content as one item is added after the other. After the animal has done so for both sets, it must then compare the two representations to choose the larger set. Previous studies based on this paradigm affirmed that animals, e.g., northern mockingbirds (*Mimus polyglottos*; Farnsworth and Smolinski, 2006), mosquitofish (*Gambusia holbrooki*; Dadda et al., 2009), non-human primates, such as rhesus monkeys (*Macaca mulatta*; Hauser et al., 2000), or chimpanzees (Beran, 2001, 2004; Beran and Beran, 2004) were able to discriminate different quantities with varying success, depending on factors such as absolute set sizes or ratios between the presented items. The problem with the two-box spontaneous choice paradigm is that while it allows exclusion of perceptually based choice it does not discriminate whether they rely on numerical or quantity information to make their choice since the number of food items is perfectly correlated with the amount of food.

The second task, the violation of expectation paradigm, also requires that animals have some mental representation of the presented items. In this task, the animals are first presented with a certain quantity and then, after it disappears, with a different quantity. If animals perceive the unexpected change, they should look longer than if no change occurred. Several species have more or less successfully solved the tasks, including rhesus monkeys (Hauser et al., 1996), mongoose lemurs (*Eulemur mongoz*; Lewis et al., 2005), cotton-top tamarins (*Saguinus oedipus*; Uller et al., 2001), or mosquitofish (Dadda et al., 2009). Lewis et al. (2005) showed that the lemurs’ performances depended on the ratio between the presented sets, e.g., that their judgment and discrimination decreased when the ratio (ratio > 0.5) increased (“Weber’s law,” in, e.g., Gallistel and Gelman, 2000). However, as shown by the different success rates, the animals might attend to different perceptual properties such as the surface area or pattern of the presented sets, requiring proper controls in order to elucidate the subjects’ quantitative skills.

Although in their natural environment both dogs and wolves have been shown to adjust their behavior in intergroup conflicts according to the number of opponents (Harrington and Mech, 1979; Sillero-Zubiri and MacDonald, 1998; Bonanni et al., 2009, 2011), little is known whether they base their choices on quantity judgments or numerical properties in such encounters. To investigate canines’ quantitative representations, several studies have been conducted. Two studies on dogs (West and Young, 2002; Ward and Smuts, 2007) and one study on coyotes (Baker et al., 2011) found positive results using the violation of expectancy looking paradigm (West and Young, 2002) as well as the two-box spontaneous choice paradigm (Ward and Smuts, 2007; Baker et al., 2011). The latter two studies showed that dogs and coyotes could discriminate between two small quantities of one to five items, if those were visible at the moment of choice and if the subjects’ performances conformed to Weber’s law. However, these results have to be considered with caution, due to (1) their use of only three very simple calculations (1 + 1 = 1; 1 + 1 = 2 and 1 + 1 = 3; West and Young, 2002), (2) decreased numerical competence if the presented quantities were invisible during the choice (Ward and Smuts, 2007; Baker et al., 2011), (3) a small sample size (*n* = 2) in the invisible choice condition in the dog study (Ward and Smuts, 2007). Furthermore, although both dogs and coyotes had to mentally compare both sets in some conditions, the discriminations could still have been based on pattern recognition, the surface area of the presented food, the volume of said food, or a combination of the above, since no controls for such confounding factors were implemented.

To further investigate canines’ competence for quantity judgment, we tested whether wolves were able to discriminate presented quantities in a two-way choice task used by Hauser et al. (2000) and adapted by Dorottya Ujfalussy (unpublished manuscript). In our study (1) the food items were placed one-by-one into an opaque container, thereby avoiding the possibility that subjects made a choice based on seeing the complete quantities of the two sets at the moment of choice, and (2) the handling time during which a smaller vs. a larger quantity would be inserted as well as the total amount of items were controlled for by adding additional stones, resulting in equal net quantities of items on both sides. We aimed at testing whether wolves could discriminate between the presented quantities when properly controlling for these perceptual properties.

## Materials and Methods

No special permission is required in Austria for using animals (wolves) in such cognitive studies. The applicatory committee for research without special permission regarding animals is the “Tierversuchskommission am Bundesministerium für Wissenschaft und Forschung (Austria).”

### Subjects

The 11 timber wolves (*Canis lupus*) that participated in this study were born in different facilities in Europe and America (see Table 1 for details). They were separated from their mothers within the first 10 days of their life, and were hand-raised and socialized at the Wolf Science Center (WSC), Austria. The animals grew up in peer groups and eight of them were introduced to packs of older animals at the age of 5 months. At the time of this study, the 11 wolves were living in three different packs in separate enclosures (2 m^2^ × 8000 m^2^ and 1 m^2^ × 4000 m^2^). The wolves were fed once or twice a week with meat or carcasses; water was available *ad libitum*. Since puppyhood all animals have regularly participated in different cognitive behavioral tests and have been trained on a daily basis. They are rewarded with dog dry food, cheese, or sausage. The training, executed by professional animal trainers, consists of obedience training, including commands such as sit, down, roll-over, or touch and is conducted either in the test building or the testing enclosure in physical separation of the pack. Accordingly, the animals are entirely used to being separated from their pack in order to work with familiar humans.

**Table 1:** **Detailed information on the subjects participating in this study**.

Subject	Origin	Litter	Pack	Age	Sex	Participation			
						Train	Test	Time	Stone
Kaspar (Ka)	Game park Herberstein, Austria	1	1	3.5	Male	x	x	x	x
Shima (Sh)	Game park Herberstein, Austria	1	1	3.5	Female	x	x	x	x
Aragorn (Ar)	Game park, Herberstein, Austria	1	1	3.5	Male	x	x	x	x
Apache (Ap)	Zoo Basel, Switzerland	2	1	2.5	Male	x	x	x	x
Cherokee (Ch)	Zoo Basel, Switzerland	2	1	2.5	Male	x	x	x	p.p.
Nanuk (Na)	Tripple D Farm, Montana, USA	3	2	2.5	Male	x	x	x	p.p.
Yukon (Yu)	Tripple D Farm, Montana, USA	4	2	2.5	Female	x	x	x	x
Geronimo (Ge)	Tripple D Farm, Montana, USA	4	2	2.5	Male	x	x	x	x
Tatonga (Ta)	Tripple D Farm, Montana, USA	5	2	2.5	Female	x	x	x	x
Kenai (Ke)	Zoo, Canada	6	3	1.5	Male	x	n.p.	n.p.	n.p.
Wapi (Wa)	Zoo, Canada	6	3	1.5	male	x	x	x	x

*Participation in the different parts of this study is included as “x” for participating, “p.p.” for participating partly and “n.p.” for not participating*.

### Experimental set-up

**Figure 1:**
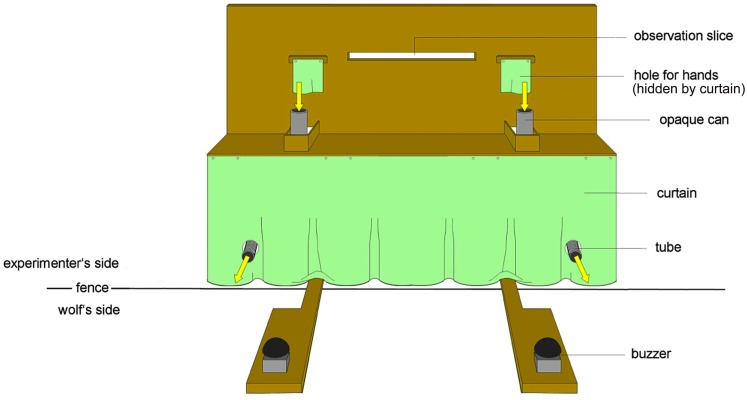
**The experimental set-up of the apparatus from the wolf’s perspective**. The draft shows the table, the buzzer and the opaque cans with the rewarding tubes, which are leading from the air lock into the testing enclosure.

The experimental apparatus was placed directly next to the fence outside of the enclosure. It consisted of a wooden table (170 cm × 40 cm × 60 cm) with two opaque cans (*h* = 14 cm, Ø = 8 cm) mounted on top (Figure 1); one to the left and one to the right side of a familiar experimenter, who was sitting on a chair behind the table opposite from the fence. The cans were fixed 5 cm from the fence and each 75 cm from the center of the table. The bottom of the cans as well as the table had a hole so that a funnel could be connected directly to each of the cans. Each funnel was further linked to a plastic tube, which led into the enclosure. In this way food that was inserted into a can could slide into the enclosure. To prevent the food from sliding into the enclosure immediately after inserting it into the can, the bottom of the cans could be closed by a plastic bar that the experimenter could remove by sliding it toward herself. Below the table, a curtain, with two holes for the tubes, prevented the wolves from seeing the lower body of the experimenter. Moreover, a visual barrier placed on the table behind the cans prevented the subject from seeing the experimenter’s upper body and, therefore, minimized the possible influence of inadvertent cues (“Clever Hans effect”; Pfungst, 1907). The visual barrier had two holes for the experimenter’s hands immediately above the cans and a slim hole at the experimenter’s eye level, which allowed her to see the cans as well as the animal’s choice. During the experiment, the experimenter wore sunglasses so that the wolves could not see their gaze.

On the wolf’s side of the fence, under each tube, a wooden panel was placed with a buzzer fixed on it.

### Basic procedure

For the experiment, the subject was separated from its pack and moved into the testing enclosure. A trainer was present in the testing enclosure, where the animal could move around freely except during the experimental trials when the trainer was holding it on a collar about 1.5 m in front of the apparatus.

The experiment started after the experimenter had taken her position and picked the required amount of food (and stones) out of a bowl on her side of the table. Both filled, closed hands were inserted into the holes above the cans. We pseudorandomized the side on which the first item was inserted, with the restriction that both sides started equally often and that the same side did not start more than three times consecutively, so as to avoid any potential side preferences. One of the items from the hand (held by two fingers, the other items hidden in the closed palm) was presented in the direction of the wolf. After the wolf looked, the experimenter placed it either onto the table next to the can (*training level 2 *+ *3 *+ *5*) or into the opaque can (*training level 4, test, time control, and stone control*). If the wolf was looking in another direction the experimenter called it by name to attract its attention. This procedure (item-by-item) was then repeated until the first hand was empty and shown to the wolf. Then the items from the other hand were placed accordingly. When both empty hands had been shown to the wolf, the experimenter gave a signal to the trainer by saying “go.”

Upon the signal, the wolf was released to make its choice. The wolves had been trained to step on the buzzer to provide an acoustic signal in order to clearly indicate their choice. In the test trials, however, a choice was regarded valid when the wolf either (1) used the buzzer, (2) stepped on the wooden panel to which the buzzer was attached to or (3) was touching the fence on the side of the can with its nose for at least 3 s. The variety of choices was necessary to avoid missing or misinterpreting the wolf’s first choice by waiting too long for it to use solely the buzzer. In the test and in both controls, the wolf only received the chosen food if the choice was correct, while in the training phase rewarding depended on the level (see below for details). The trainer called the animal back after it was rewarded or saw that it did not get a reward.

Depending on the experimental phase (training, test, controls), the experimenter pulled out the plastic bar after a wolf’s correct choice and thus released the reward that slid into the enclosure. If the items were placed on the table, the experimenter picked them up and inserted them into the can from where they could slide into the enclosure. When the wolf chose incorrectly, the experimenter retrieved the items from both sides.

Three professional animal trainers were involved in this experiment shaping the buzzer pressing behavior of each subject during the training phase and handling the wolves during the experiments. During the experiments, the trainers wore a baseball cap and looked down at their feet preventing them from seeing the placement of the food.

Cheese (sort: Gouda; 1 cm × 1 cm × 1 cm) was used to reward the animals to guarantee high motivation to work. For the controls (see below), black stones of comparable size were used. We conducted only one session per day with 1–2 days (test and control phase) elapsing between sessions.

### Detailed procedures

We conducted a training phase, a testing phase, and two control experiments (time and stone control; Table 2).

**Table 2:** **Conditions of the experiment and criteria for the training steps**.

Condition	Aim (criterion for each training step)	Items	*N*
Training step 1	Confident buzzer usage (10x sequentially per session)	Dry dog food	11
Training step 2	Making a choice (9/11 trials)	Cheese	11
Training step 3	Discrimination of stone and cheese (6/7 trials)	Cheese, stone	11
Training step 4	Introduction opaque can, stone vs. cheese (6/7 trials)	Cheese, stone	10
Training step 5	Repetition choice for more items (6/7 trials)	Cheese	10
Quantity test	Quantity discrimination	Cheese	10
Time control	Handling time adaption	Cheese, stones	10
Stone control	Dismissal of time and sound factor	Cheese, stones	10 (8)

*One of the 11 animals did not reach the criterion of *step 4* and, therefore, did not participate in the rest of the experiment. In the stone control 10 animals participated in three of the four sessions and eight of them participated in all four sessions*.

#### Training phase

The training phase was conducted to familiarize the animals with the apparatus and the procedure. It consisted of five steps: (1) buzzer training to teach the wolf how to use the experimental apparatus, (2) choosing the larger of two visible quantities (1 vs. 4) presented on the table, (3) discrimination between a visible piece of stone and a visible piece of cheese, (4) same as *step 3*, but now the food and stone were inserted into the opaque cans and thus were invisible during the choice and (5) choosing once again the larger of two visibly presented quantities (1 vs. 4) to assure that the wolves still chose the larger number after training *step 3 and 4*.

In *step 1*, the animals were trained using a clicker (operant conditioning with a secondary reinforcer) and dry dog food to push the buzzer with their paw. No table was present and the rewarding was done by hand. First, only one buzzer was available to train the animals how to operate it with their paws. After the wolves were able to push the buzzer on the command “touch” with the paw 10 times in a row, the second buzzer was introduced. *Step 1* was continued till the wolves could use both buzzers showing no side preference. That is pressing the buzzer on the side the trainer pointed to at least 10 times in a row in one session. The number of trials per day depended on the motivation and concentration of each animal and thus varied between sessions (range: 7–15).

In *step 2*, the wolves were trained to choose the larger of two quantities (four against one) by placing cheese pieces next to the opaque cans on the table in full view of the wolves. To avoid a situation in which the wolves would choose based on other potential factors like side preference or order of placement rather than quantity, we presented the four possible combinations (*R* – 1 vs. 4 (= four pieces placed first on the right side, then one piece placed on the left side), *R* – 4 vs. 1, *L* – 1 vs. 4, *L* – 4 vs. 1) in a randomized and predetermined order in each session. In *step 2*, we conducted eight trials per session if each choice was correct. However, if the subject made a mistake, the same combination was repeated until the animal chose the larger reward (correction trials) and thus the number of trials per session increased. The criterion to pass *step 2* was at least nine correct choices in the last 11 trials to assure that the animals made correct choice at least twice in each of the four possible combinations.

In *step 3*, the wolves had to discriminate between one piece of cheese (ch) and one stone (st) that were placed on the table in full view. We conducted seven trials per session and the animals had to choose the cheese at least six times in one session in order to reach criterion. A milder criterion was used than in *step 2* to keep habituation to the presence of the stone at minimum, and thus, to avoid that the wolves learn to base their decision on discrimination of the stimuli instead of using cognitive processes (Stevens et al., 2007).

In *step 4*, we inserted the food and the stone into the opaque cans requiring the wolves to make a choice when neither cheese nor stone were visible. Each session consisted again of seven trials, and the same criterion was used as in *step 3*. In *step 3 and 4*, we always released the selected item into the enclosure to give the animals the opportunity to inspect the stone. The stones were collected by the trainer and handed over to the experimenter at the end of each trial.

*Step 5* was similar to *step 2* with the only difference that no correction trials were conducted limiting each session to seven trials. The criterion was set at six correct choices in the last seven trials.

For each step, we counted the number of trials a wolf needed to reach the criterion (including the correction trials in *step 2*).

#### Testing phase and control experiments

##### Quantity discrimination test

Using the opaque cans, we tested whether the animals could also discriminate between the six pairs of quantities (1 vs. 2, 1 vs. 3, 1 vs. 4, 2 vs. 4, 2 vs. 3, 3 vs. 4) not tested in the training phase. Depending on the combination, the distance and the ratio between the two presented quantities varied. Randomizing the side and the placing order, each pair can be presented in four different ways resulting in 24 conditions. All of these conditions were repeated twice in a total of eight test sessions of six trials each.

##### Time and stone control

**Figure 2:**
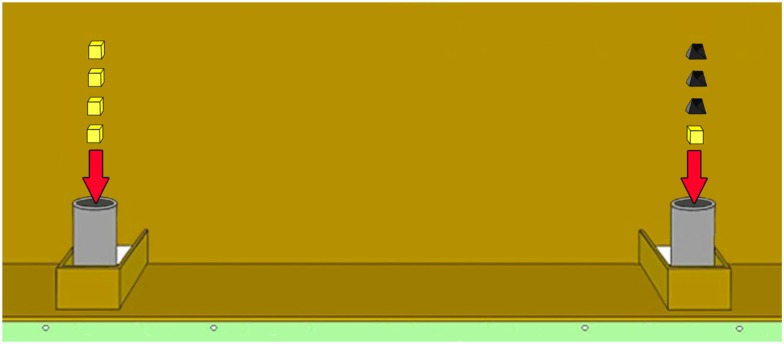
**Illustration of the task 4 vs. 1 in *Time control***.

**Figure 3:**
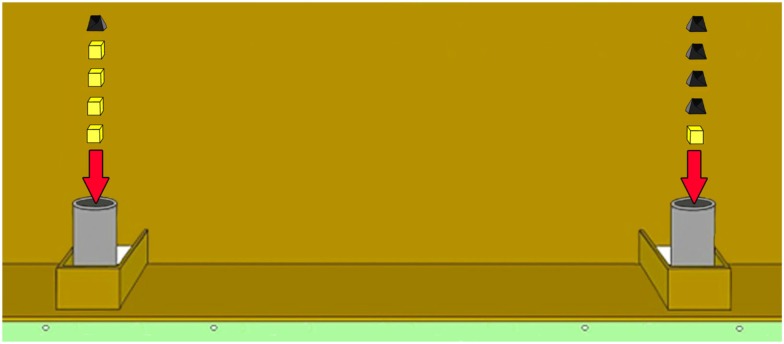
**Illustration of the task 4 vs. 1 in *Stone control***.

We conducted two control experiments after the quantity discrimination test to stepwise exclude further factors that might have had an influence on the test performance. Each control experiment consisted of four sessions of six trials each. Both controls contained the following three of the six quantity pairs used in the test: 1 vs. 2, 1 vs. 4, 2 vs. 3. Accordingly, we had a set with a small distance and an intermediate ratio between sets (1 vs. 2), a set containing a large distance and a small ratio between both sets (1 vs. 4) and a set with a large ratio and an intermediate distance (2 vs. 3). The first control was conducted to investigate whether the wolves solved the discrimination task by actually comparing the food quantities or, alternatively, by using the time interval it took to insert the different number of food pieces into the cans (time control). Accordingly, we added stones to the smaller quantity of cheese pieces until both cans contained the same number of items – that is the handling time was the same on both sides (Figure 2). However, since the stones were always added last to the side with the fewer pieces of cheese, it was still possible that the animals solved this first control experiment by avoiding the (sound of the) stone(s). To exclude this opportunity we added an extra stone to both sides in the stone control experiment (e.g., 4 vs. 1: one can contained four pieces of cheese and one stone and the other can containing one piece of cheese and four stones; Figure 3).

Each session of the test and both control experiments were followed by a so-called concentration control. The latter consisted of four trials in which only one piece of cheese was inserted into one of the two cans randomizing the sides. This concentration control was conducted to ensure that the animal paid attention to the experiment and did not solely pick a side randomly. All of the participating animals performed without a single mistake in the concentration controls of the test and both control experiments.

### Data analysis

We first examined whether non-quantity factors influenced the performance of the animals in the testing phase or control experiments by calculating non-linear mixed effect models (nlme) using a binomial distribution. Accordingly, we analyzed whether the wolves’ choices for the cans were influenced by the side with the larger quantity of food items (“side_larger quantity), by the order of placing (“order_first” and “order_second”; large amount first or second) or the session (“sess”; 1–8). To test for side bias, bias for side of first presentation and changes in performance across all trials we compared the data to chance level with a one-sample *t*-test each. Provided those non-quantity factors had no influence, they were excluded from further analyses. In the main analyses we examined if the animals’ choice of the larger quantity depended on the ratio of the two numbers presented (“ratio”; 0.25, 0.33, 0.5, 0.66, 0.75). Wolf identity and the sessions were treated as random factors in the models. The data analysis was repeated excluding the combinations (1 vs. 4 and 4 vs. 1), since these were extensively used in the training phase. For investigating the performance of each individual (No. of correct choices) in the test, we additionally conducted a binomial test. When an individual wolf did not choose the larger quantity above chance, we analyzed whether its performance, respectively its success rate (“choice_larger_quantity,” correct choice of the side with the larger quantity of cheese), was influenced by the order of placing, side of larger quantity and if it varied across the sessions. The control experiments were analyzed with the same model as the quantity discrimination test.

To control for training effects in all three tests we calculated a nlme using a binomial distribution to investigate whether the performance was influenced by the number of training trials (“train,” overall number of trials to complete all training sessions). Treatment (“treatment_test,” “treatment_time_control,” and “treatment_stone_control”), wolf identity and the sessions were included as random factors. The wolves might have learned also across the test and control trials, predicting an increasing performance (“choice_larger_quantity”) from the quantity discrimination test to time control and stone control. We tested this by running a nlme using a Poisson distribution and analyzing whether the choice was influenced by the treatment (*test, time control, stone control*), the session (1–8), or trial (*test:* 1–6, *time control*, *stone control*: 1–4).

The data were analyzed using the statistical program *R* (version 2.14.1). Results are given for two-tailed tests and alpha was set at 0.05. Trends are reported for 0.1 < *p* < 0.05.

## Results

### Training phase

Ten of the 11 wolves participating in this study passed all training steps and were tested in the quantity discrimination test. The subjects needed between 150 and 404 trials to pass the training and proceed to the test phase (Table 3). One wolf (Kenai) did not reach the criterion of *step 3* (discrimination of stone vs. cheese, visible) and, therefore, did not proceed to the next step.

**Table 3:** **Number of trials every subject needed to reach the next step (*step 1*–*5*) in the training phase**.

Subject	*Step 1*	*Step 2*	*Step 3*	*Step 4*	*Step 5*	Overall
Apache	85	14	7	7	113	226
Aragorn	115	16	14	21	166	332
Cherokee	107	11	7	6	131	262
Geronimo	70	7	7	6	90	180
Kaspar	106	12	21	6	145	290
Kenai	72	22	56	n.p.	n.p.	dism.
Nanuk	132	49	7	14	202	404
Shima	91	10	7	6	114	228
Tatonga	99	7	7	21	134	268
Wapi	106	11	21	7	145	290
Yukon	47	15	6	7	75	150

*The “n.p.” stands for steps in which a subject was not participating because it did not reach the criterion. Not passing a step leads to not proceeding in the quantity discrimination test and being dismissed for the rest of the study (dism.)*.

### Testing phase

#### Non-quantity factors

The animals’ choices in the test were influenced neither by the placing order (large amount first or second; NLMEorder: *t*_394_ = −0.07, *p* = 0.93) nor the session (NLMEsess: *t*_69_ = −0.38; *p* = 0.70). Further on, although the wolves chose the right can more often than the left can if the larger amount of cheese was placed second (NLMEorder: *t*_396_ = 2.814, *p* = 0.005), no side bias occurred (one-sample *t*-test: *t*_9_ = −0.64, *p* = 0.53).

In the time and stone control, we again found no differences in performance across sessions (NLMEsess: time control: *t*_29_ = −1.10; *p* = 0.27; stone control: *t*_27_ = 0.76; *p* = 0.45), and the one-sample *t*-test revealed no side bias in either control (time control: *t*_9_ = −0.60, *p* = 0.56, stone control: *t*_9_ = 0.11, *p* = 0.91). While the placing order (large amount first or second) had no significant effect on the animal’s choices in the time control (NLMEorder: *t*_196_ = 0.19, *p* = 0.84), it had an influence in the stone control (NLMEside_larger_quantity _×_ order: *t*_186_ = 2.401, *p* = 0.017), suggesting that the wolves chose more often the larger quantity if it was placed second. However, in both situations (larger quantity being placed first or second) they chose the larger quantity more often than the smaller one (NLMEside_larger_quantity: placed first: *t*_77_ = 2.425; *p* = 0.018; placed second: *t*_72_ = 4.980; *p* < 0.001).

#### Quantity discrimination test

**Figure 4:**
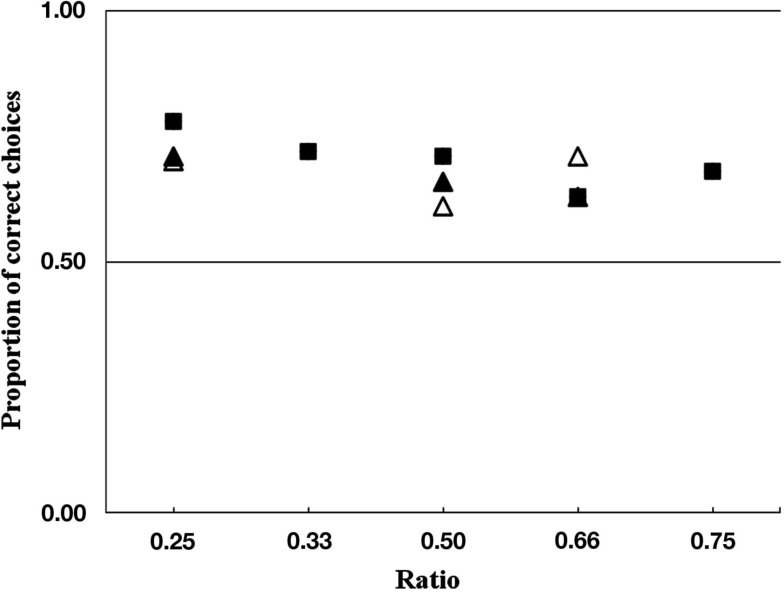
**Performance of the wolves across all conditions (■ = quantity test, ▲ = time control, △ = stone control) shown as proportion of correct choices for the given ratios**. The ratio 0.33 and 0.75 were only present in the quantity test.

Overall the wolves chose the side with the larger quantity above chance in 70.21% of the cases (337 of 480 trials; Table 3; one-sample *t*-test: *t*_9_ = 8.881, *p* < 0.001). After excluding the combinations 1 vs. 4 used extensively in the training, we found that the wolves still chose more often the larger quantity in 69% of the cases (276 of 400 trials; one-sample *t*-test: *t*_9_ = 8.249, *p* < 0.001). There was a tendency for improved performance as the ratio between sets got lower (NLME_ratio_: *t*_471_ = −1.71, *p* = 0.08; Figure 4).

At the individual level, nine out of the 10 wolves picked the side with more pieces of cheese above chance. The wolf Nanuk had the most correct choices with 39 of 48 trials choosing the larger quantity (81.25%; binomial test: *p* = < 0.001). One wolf (Kaspar) was not choosing the big amount more often than the small one (binomial test: *p* = 0.19). However, his choice to take the larger quantity depended on whether the larger quantity was placed first or second (NLMEorder: *t*_39_ = −2.097, *p* = 0.043).

#### Time control and stone control

With 160 of 240 (66.67%) correct choices in the time control, the group chose more often the side with the larger amount (one-sample *t*-test: *t*_9_ = 5.164, *p* = 0.001). The performance of the wolves was not influenced by the ratio between the two presented sets (NLME_ratio_0.5_: *t*_198_ = −0.73, *p* = 0.46; _ratio_0.066_: *t*_198_ = −1.11, *p* = 0.26). At the individual level, in the time control two wolves chose more often the side with more pieces of cheese (binomial test: Apache: *p* < 0.001; Aragorn: *p* = 0.023) and two other wolves showed a tendency to do so (binomial test: Geronimo: *p* = 0.06; Yukon: *p* = 0.06).

In the stone control, the wolves chose the larger quantity of cheese in 67.11% of the trials (135 out of 228 trials; one-sample *t*-test: *t*_9_ = 4.391, *p* = 0.002). We found that the wolves made their choice independent from the ratio between the two sets (NLME_ratio_0.5_: *t*_187_ = −0.13, *p* = 0.17; _ratio_0.66_: *t*_187_ = 0.16, *p* = 0.87; Figure 4). At the individual level, three animals chose significantly more often the larger amount of cheese (binomial tests: Apache: *p* = 0.002; Aragorn: *p* = 0.023, Tatonga: *p* = 0.007), and two others showed a tendency for the larger quantity (binomial test: Nanuk: *p* = 0.096; Geronimo: *p* = 0.064).

### “Training effect” and “learning effect”

**Figure 5:**
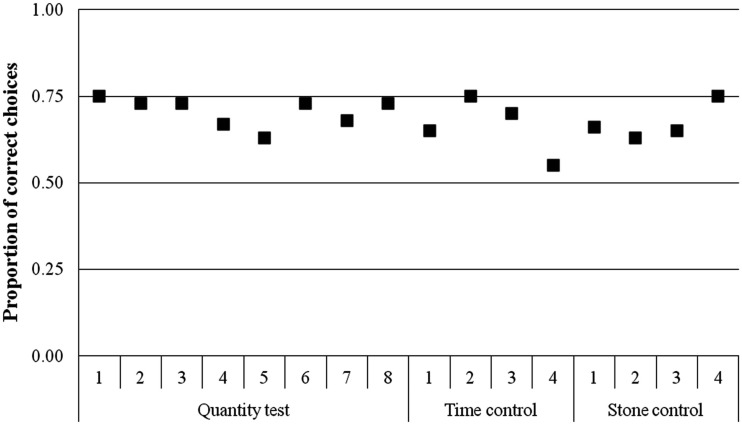
**Performance of the wolves given as the proportion of correct choices for every session across the different experimental conditions (Quantity test: 1–8, Time control: 1–4, Stone control: 1–4)**.

The number of training trials needed to reach the testing phase did not have any influence on the frequency of choosing the larger quantity (NLMEtrain, *t*_8_ = 1.02, *p* = 0.33). This pattern (“Training effect”) did not differ between the tests and control sessions (NLMEtrain _×_ treatment_test: *t*_16_ = 1.47, *p* = 0.16; train _×_ treatment_control: *t*_16_ = 0.42, *p* = 0.67). Regarding “learning effect” throughout the testing periods, we found that the wolves’ performance did not change (NLMEsess: *t*_936_ = −1.16, *p* = 0.25). Furthermore, the wolves’ performances did not differ between the test and control sessions (NLMEsess _×_ treatment_test: *t*_143_ = 0.93, *p* = 0.35; sess _×_ treatment_control2: *t*_143_ = 1.39, *p* = 0.16; Figure 5).

## Discussion

We found that the wolves’ choices were not influenced by the side and order of placement or the session, suggesting that the wolves based their choice on the representation of two food quantities. Nine of 10 wolves performed significantly above chance also at the individual level. Two additional controls assured that the animals did not use the handling time (time control) or the sound of the stone(s; stone control) as discriminative stimuli by stepwise excluding these factors. In the controls, three animals still chose significantly more often the larger quantity of cheese and two other wolves showed a tendency to do so.

In Ward and Smuts (2007) study, two dogs successfully discriminated two presented quantities even if they were not visible at the time of choice. Baker et al. (2011) showed that coyotes behaved similarly to dogs, but could only discriminate correctly between two quantities if the sets were visible at the moment of choice. Our paradigm of presenting the food items invisibly controlled for some of the confounding properties (volume, surface area, pattern recognition) that were not controlled for in the coyote study. Moreover, by adding the first control situation, we equalized not just the handling time, but also the total amount of items that were inserted into each can, making it much harder for the animals to choose based on properties other than the quantity of food. However, it is theoretically still possible that the animals made their choice based on the total amount of cheese that was added to each can, assuming that they left the inedible stones out from their representation of the total amount of food.

The wolves’ performance did not improve with decreasing ratio of the two sets, and thus did not confirm to Weber’s law (Gallistel and Gelman, 2000). However, in the testing phase the wolves showed a tendency to improve in discriminating both sets when the ratio was decreasing. In previous studies, two dogs failed to discriminate correctly in the 3 vs. 4 task (ratio = 0.75) and only one of them was able to discriminate 2 vs. 3 (ratio = 0.66; when not visible at the moment of choice; Ward and Smuts, 2007). Moreover, coyotes experienced trouble discriminating the visible sets of 2 vs. 3 and 3 vs. 4 at the group level, whereas they reliably chose the larger of two presented sets when the ratio decreased below 0.5 (e.g., 1 vs. 4., 1 vs. 3, and 2 vs. 5). Those results are in support of Weber’s law and suggest that the performance of canines might decrease or even break down for high ratios (ratio > 0.5). On the other hand, theoretically Weber’s law is mainly connected to larger set sizes (above four items). That is, it is interesting that these previous canine studies found limitations in the animals’ performance for small quantities. The wolves in our study successfully discriminated all combinations above chance, with a slight tendency for being better with sets of a smaller ratio in the test. Interestingly, however, in both controls the set ratio had no influence on the performance. A possible explanation might be that the wolves used the different handling times or the total amount of all items as indices for the larger quantity in the first test condition whereas they could rely only on food quantity in the control conditions. This would mean that instead of benefiting from multiple sources of information, the wolves could profit more from clear and unequivocal information provided in the control conditions.

Although the performance of the wolves was not influenced by the set ratio in the control conditions, it is not possible to confirm which model explains the numerical skills of wolves (small quantities: object-file model’ (e.g., Kahneman et al., 1992; Hauser et al., 2000; large quantities conforming to Weber’s law: accumulator model (e.g., Meck and Church, 1983; Gallistel and Gelman, 2000). To confirm which model would be better suited one would need to demonstrate similar results with higher ratios and larger set sizes (e.g., one to seven pieces of cheese). However, expanding the set size could prove difficult because based on the experience in this study, the wolves’ concentration decreased with increasing number of items. In the *stone control* an extra stone was added to both sides and, therefore, the total number of items on each side was increased to five pieces of cheese and stones. Some of the wolves seemed to get over-excited or even frustrated (increased locomotion: strong pulling toward the table or jumping around) because of the longer handling time, which might have an influence on the performance if even bigger numbers are used.

Studies on other species that used a comparably complex method could show that both monkeys (Beran, 2007) and great apes (Hanus and Call, 2007) are able to discriminate quantities that are presented item-by-item depending on the ratio between two sets. In both studies they tested different combinations between one and 10 items and found that the animals’ performance decreased with increasing ratio. This is in curious contrast with our results on the wolves. Beran (2007) tested two rhesus monkeys and found that the animals failed in discriminating high ratios (>0.83). Further, both animals failed to discriminate sets of a ratio of 0.6 and only one animal was able to choose the larger quantity when the ratio was 0.75. The latter conforms to our 3 vs. 4 combination that the wolves were able to discriminate above chance. Additionally, Beran (2007) showed that when the total set presentation could not be used as a cue (e.g., by varying the presentation time of smaller and larger sets) then the animals’ performance fell to chance level. In contrast to this, in our wolf study, we equalized the duration by the addition of stones, excluding this potential non-numerical influence, and still found that the wolves performed above chance.

Hanus and Call (2007) tested different great apes and showed that after item-by-item presentation, at group level all species except bonobos (but overall only 26% of the subjects) selected the larger quantity in low quantity combinations (up to six) in accordance with Weber’s law. However, Hanus and Call (2007) did not conduct any control experiments to exclude non-numerical influences such as duration of handling. Therefore, it can not be excluded that the performance of the subjects relied on these cues, and it is possible that – similarly to the wolves – they would have performed better if they could discriminate the combinations purely based on food quantities.

In summary, our study showed that wolves are able to make quantitative judgments even when alternative strategies such as paying attention to non-numerical properties such as the surface area or time and total amount are ruled out. To determine whether and when their quantity discrimination conforms to Weber’s law and to elucidate which model describes the numerical skill of wolves best, studies using larger quantities are needed. Finally, to compare their performance with that of other species, better controlled comparative experiments are necessary.

## Conflict of Interest Statement

The authors declare that the research was conducted in the absence of any commercial or financial relationships that could be construed as a potential conflict of interest.
